# A Pediatric Patient with Intravenous Cyclosporine Anaphylaxis Who Tolerated the Oral Form

**DOI:** 10.4274/tjh.2014.0206

**Published:** 2014-12-05

**Authors:** Pamir Işık, Namik Özbek, Emine Dibek Mısırlıoğlu, Turan Bayhan, Suna Emir, Fatih Mehmet Azık, Bahattin Tunç

**Affiliations:** 1 Ankara Children’s Hematology and Oncology Education and Research Hospital, Clinic of Pediatric Hematology, Ankara, Turkey; 2 Ankara Children’s Hematology and Oncology Education and Research Hospital, Clinic of Pediatric Allergy, Ankara, Turkey; 3 Hacettepe University Faculty of Medicine, Division of Pediatric Hematology, Ankara, Turkey

**Keywords:** cyclosporine, Toxicity, stem cell transplantation

## TO THE EDITOR

Cyclosporine is a potent immune suppressant and prevents T-cell activation and graft-versus-host disease by inhibiting calcineurin phosphatase [1,2]. Anaphylactic reaction due to intravenous infusion is a rare side effect, reported in 0.1% of patients [3,4]. Cremophor EL (polyoxyethylated castor oil), a solubilizing agent of the parenteral cyclosporine, has been blamed for hypersensitivity reactions [5]. Here we report a patient who developed anaphylaxis due to an intravenous form of cyclosporine.

A 17-year-old boy, diagnosed with relapsed T-cell lymphoblastic lymphoma after achieving complete remission, underwent bone marrow transplantation from his fully matched brother. Intravenous cyclosporine (3 mg/kg/day) was started at day -1. At day +10, he developed a maculopapular rash on his trunk, fever, and weight gain, diagnosed as engraftment syndrome. His symptoms improved with intravenous methylprednisolone treatment (2 mg/kg/day). At day +14, at 10 min after the start of cyclosporine infusion, he developed disseminated erythematous rash, respiratory distress, severe chest pain, and facial edema. His systolic blood pressure was within the normal range, but his pulse rate decreased to 50/min. Cyclosporine infusion was stopped and adrenaline, methylprednisolone, and pheniramine were intravenously administered. His complaints resolved within 30 min. We concluded that this reaction had occurred against the castor oil that exists in the intravenous form. Since cyclosporine treatment was crucial, we decided to give a capsule formulation that did not contain castor oil (Panosporin®). The capsule was given in a 3-dose graded challenge (25 mg, 25 mg, and 50 mg with 30-min intervals) without significant reaction. In the following period he received scheduled doses without any problem. Skin tests performed with intravenous cyclosporine 5 months after anaphylaxis revealed positive results in our patient, but the test was negative for the donor. Informed consent was obtained.

Polyoxyethylated castor oil has been associated with severe anaphylaxis [6]. A recent report concerning cyclosporine-induced anaphylaxis revealed that 11 patients had a reaction with the intravenous form. Seven of these patients tolerated an oral formulation of cyclosporine. It was claimed that hypersensitivity reaction to one formulation of cyclosporine does not preclude the use of a different formulation [5]. Some patients in the literature were reported to have allergic reactions to a parenteral form of both cyclosporine and tacrolimus attributed to castor oil, which is present in the formulation of both drugs [7,8]. Those patients had tolerated the oral formulation of cyclosporine without castor oil, as did our patient. Since the oral form of Panosporin® does not contain castor oil, we chose this drug for treatment for our patient without further reactions.

Proposed mechanisms related to allergic reactions due to polyoxyethylated castor oil include IgE-mediated hypersensitivity, complement activation, and mast cell degranulation [5]. Our patient had a positive intradermal test for intravenous cyclosporine including polyoxyethylated castor oil. The positivity of the intradermal test suggested an IgE-mediated reaction. Interestingly, intensive immune suppression in bone marrow recipients may facilitate the rare reaction to castor oil [7]. In conclusion, in the case of anaphylaxis with the parenteral form, oral products of cyclosporine that do not contain castor oil can be tried in patients who underwent bone marrow transplantation.

**Conflict of Interest Statement**

The authors of this paper have no conflicts of interest, including specific financial interests, relationships, and/or affiliations relevant to the subject matter or materials included.
